# Pediatric Sequential Organ Assessment Score: A Comprehensive Review of the Prognostic Marker in the Pediatric Intensive Care Unit

**DOI:** 10.7759/cureus.60034

**Published:** 2024-05-10

**Authors:** Aashita Malik, Amar Taksande, Revatdhamma Meshram

**Affiliations:** 1 Department of Pediatrics, Jawaharlal Nehru Medical College, Datta Meghe Institute of Higher Education and Research, Wardha, IND

**Keywords:** psofa score, picu, pediatrics, morbidity, mortality

## Abstract

Critically ill children admitted to the pediatric intensive care unit (PICU) face a substantial risk of morbidity and mortality, regardless of whether they are in developed or developing countries. To aid in treatment planning, various prognostic scoring systems have been developed to predict the likelihood of morbidity and death in these young patients. While the sequential organ failure assessment (SOFA) score has been validated as an independent risk predictor for adult mortality in cases of confirmed or suspected sepsis, it is not suitable for use in children due to its lack of age normalization. Children in critical condition often exhibit significant deviations from the normal physiological balance of their bodies. These deviations from the typical range of physiological variables can be leveraged to estimate the extent of these variations and create scoring systems. In this context, the pediatric SOFA (pSOFA) score was developed by modifying the original SOFA score and incorporating age-adjusted cutoffs for various bodily systems. The objective of this review is to assess the effectiveness of the pSOFA score in predicting sepsis-related mortality in pediatric patients within the PICU setting.

## Introduction and background

The pediatric intensive care unit (PICU) serves as the last line of defense against the substantial risk of morbidity and mortality faced by critically ill children, regardless of geographical boundaries. In the challenging landscape of pediatric critical care, one of the paramount objectives is to predict and manage the unpredictable, namely, the outcome of these young patients. To this end, numerous prognostic scoring systems have been devised to decipher the likelihood of morbidity and mortality. While the sequential organ failure assessment (SOFA) score has been a steadfast companion in predicting adult mortality, particularly in cases of confirmed or suspected sepsis, it leaves a void in the realm of pediatric care due to the absence of age normalization. Indeed, children in critical condition often manifest significant deviations from the norm in terms of their physiological equilibrium. These variations in physiological parameters, veering away from the usual range, hold the key to estimating the extent of these deviations and serve as the foundation for constructing scoring systems tailored to the pediatric population. In response to this need, the pediatric SOFA (pSOFA) score emerged as an adaptation of the original SOFA score, equipped with age-adjusted thresholds for various bodily systems. It represents a promising tool in the arsenal of pediatric critical care, poised to enhance our ability to foresee sepsis-related mortality within the PICU setting. In a lot of recent studies, it has been seen that SOFA score at admission is useful for predicting outcomes in the general PICU population and is more accurate than systemic inflammatory response syndrome (SIRS) for the definition of pediatric sepsis [1].

This comprehensive review endeavors to delve into the depths of the pSOFA score, its origins, and its clinical utility. We will explore its effectiveness as a prognostic marker in the PICU, shedding light on its role in predicting the outcomes of critically ill pediatric patients, especially in the context of sepsis. As we navigate this terrain, we aim to uncover the nuances, strengths, and limitations of the pSOFA score, ultimately contributing to our understanding of its significance in the intricate web of pediatric critical care.

## Review

Critically ill children admitted to the PICU face a substantial risk of both morbidity and mortality, regardless of whether they are in developed or developing countries [1]. Despite the various underlying illnesses, there is a lack of comprehensive data regarding the specific factors contributing to mortality in these cases. A deeper understanding of these variables is crucial for conducting thorough assessments of these patients and identifying areas in therapy and research that require attention to improve their short- and long-term outcomes. Pediatric patients in critical condition often experience significant deviations from the typical physiological balance of the body. These shifts in physiological variables away from the normal range can be harnessed to estimate the extent of these variations, and the deviations in these changing variables can be used to develop scoring systems [2].

In order to provide severely ill children with the demanding and necessary care they need, the PICU is crucial. Children in ICUs face a significantly increased risk of morbidity and death, in both developed and poor nations. The number and quality of PICUs are rising in developing nations like India, but the process is still uphill because the units require a sizable and highly skilled workforce in addition to pricey, sophisticated equipment. Therefore, it takes time to implement strategies, tactics, and scoring systems that predict the risk of death and morbidity in these patients. This enables these systems to support prompt and targeted decisions about the allocation of various specialties and resources, resulting in extremely fruitful outcomes [3].

Sepsis stands as a significant contributor to illness and death in children, emphasizing the critical importance of early detection. Timely identification of sepsis allows for interventions such as fluid resuscitation and prompt administration of antibiotics, ultimately preventing morbidity and mortality. The definition of sepsis has changed over time, with a growing emphasis on defining it based on organ dysfunction rather than a systemic inflammatory response.

In particular, the Third International Consensus Definitions for Sepsis and Septic Shock (Sepsis-3) underscores sepsis as a combination of four key factors: (1) a life-threatening condition, (2) organ dysfunction, (3) an imbalanced host response, and (4) the presence of a highly suspected or documented infection. This approach provides a more nuanced understanding of sepsis, guiding healthcare professionals in more accurately identifying and addressing the condition in pediatric patients [4]. Various prognostic scoring systems, such as the Pediatric Index of Mortality (PIM and PIM2), Pediatric Risk of Mortality (PRISM, PRISM3), SOFA, pSOFA score, and the Pediatric Logistic Organ Dysfunction (PELOD) score, have been created to anticipate morbidity and mortality in children within the PICU. These tools play a crucial role in treatment planning, offering valuable insights for healthcare professionals in predicting and managing the outcomes of critically ill pediatric patients [5]. Over the last three decades, various scoring systems tailored for ICU patients have been developed. These systems utilize regularly collected patient-specific data, allowing for an assessment of disease severity and providing an estimate of in-hospital mortality [6]. Schlapbach et al. demonstrated that adapting Sepsis-3 to age-specific criteria performs better than Sepsis-2-based criteria [7].

Considering the incorporation of scores like PIM and PIM2, PRISM and PRISM3, SOFA, pSOFA score, and the PELOD score as indicators of organ dysfunction in infected children could be contemplated to align with the Sepsis-3 definitions for pediatric patients. However, it is important to note that the distinct range, scale, and coverage of these scores significantly differ from the SOFA score, posing challenges in their simultaneous application [8]. In the realm of critical care, the SOFA score system is commonly employed to identify individuals requiring prompt intervention and to promptly identify the onset of sepsis [9].

However, the SOFA score was previously validated as an independent risk factor of adult mortality associated with confirmed or suspected sepsis. However, the SOFA score was not normalized against age, thereby making it unsuitable for children [10]. Having different definitions of sepsis for patients above or below the pediatric-adult threshold does not have any physiologic justification and should be avoided [8].

The pSOFA score was created by adjusting the original SOFA, incorporating age-specific cutoffs for the cardiovascular subscore (mean arterial pressure) and renal subscore (serum creatinine level). Additionally, modifications were made to the respiratory subscore, which now considers the ratio of peripheral oxygen saturation to fractional inspired oxygen (SpO2-FIO2) as a noninvasive indicator of lung injury (Table 1) [8,11].

**Table 1:** Pediatric sequential organ failure assessment score ICU: Intensive care unit; IQR, interquartile range; LOS: length of stay; OD: organ dysfunction; PRISM3: Pediatric Risk of Mortality 3; pSOFA: pediatric sequential organ failure assessment (a) Clinical variables included demographic, clinical, and microbiological characteristics of patients with confirmed or suspected infection and with or without sepsis according to the Sepsis-3 definitions. (b)Continuous variables are presented as median (IQR) values. (c) Other hospitals included other emergency departments. (d) More than one type is possible, and percentages may add to more than 100%. (e) Ventilator-free, vasoactive infusion-free, and ICU-free days were calculated using 28 days

Variables		Score				
		0	1	2	3	4
Respiratory	PaO2:FiO2	>400	300-399	200-299	100-199 with respiratory support	<100 with respiratory support
	SpO2:FiO2	>292	100-149	50-99	20-49	<20
Coagulation	Platelet count	>150	100-149	50-99	20-49	<20
Hepatic	Total bilirubin	<1.2	1.2-1.9	2-5.9	6-11.9	>12
Cardiovascular: Map by age group or vasoactive infusion	<1m	>46	<46	Dopamine <5 or dobutamine any dose	Dopamine >5 or epinephrine <0.1 or norepinephrine <0.1	Dopamine >15 or epinephrine >0.1 or norepinephrine >0.1
	1-11 m	>55	<55			
	12-23 m	>60	<60			
	24-59 m	>62	<62			
	60-143 m	>65	<65			
	144-216 m	>67	<67			
	>216 m	>70	<70			
Neurologic	Glasgow coma score	15	13-14	10-12	6-9	<6
Renal: Creatinine by age group	<1 m	<0.8	0.8-0.9	1.0-1.1	1.2-1.5	>1.6
	1-11 m	<0.3	0.3-0.4	0.5-0.7	0.8-1.1	>1.2
	12-23 m	<0.4	0.4-0.5	0.6-1.0	1.1-1.4	>1.5
	24-59 m	<0.6	0.6-0.8	0.9-1.5	1.6-2.2	>2.3
	60-143 m	<0.7	0.7-1.0	1.1-1.7	1.8-2.5	>2.6
	144-216 m	<1.0	1.0-1.6	1.7-2.8	2.9-4.1	>4.2
	>216 m	<1.2	1.2-1.9	2.0-3.4	3.5-4.9	>5
Total score						

Comparison of pSOFA in terms of sensitivity and specificity

Various scoring systems for pediatric organ dysfunction, such as the PELOD score, the updated PELOD-2 score, and the Pediatric Multiple Organ Dysfunction Score, consider the age-specific nature of their variables. El-Mashad et al. conducted an evaluation of the age-adjusted SOFA score's performance in children admitted to the PICUs [1]. The study aimed to determine whether the SOFA score could rival the SIRS in diagnosing sepsis, following the recommendations in the Sepsis-3 consensus definitions.

Results indicated that the SOFA score was significantly higher in nonsurvivors (p < 0.001), and mortality rates increased progressively across patient subgroups with lower to higher SOFA scores. The SOFA score upon admission proves valuable in predicting outcomes within the general PICU population and exhibits greater accuracy than SIRS in defining pediatric sepsis. Jyotsna et al. conducted a study to assess and compare the severity, disease progression, and outcomes of critically ill children admitted to the PICU using various scoring systems [2]. Two hundred children aged one month to 14 years were enrolled in the study. Prognostic scoring systems, namely, PRISM4 and PIM3, were employed to evaluate outcomes, mortality rates, and the duration of PICU stays. In contrast, PELODS and pSOFA were descriptive scores used to assess multiorgan dysfunction [2].

The study revealed a significant positive correlation between mortality and scores on the first day of admission, specifically PRISM4, PIM3, PELOD2, and pSOFA (p < 0.00001). Notably, pSOFA and PELOD2 demonstrated superior discrimination power, as indicated by higher area under the curve (AUC) values of 0.77 and 0.74, respectively. The findings led to the conclusion that pSOFA and PELOD2 scores serve as reliable predictors of mortality in critically ill children. Sun et al. reported SOFA being superior to SIRS for predicting mortality in PICU patients with sepsis [12]. The pSOFA score, an age-adjusted pediatric version of the adult SOFA score, was adapted and validated by Matics et al. and used it to evaluate the Sepsis-3 definitions in critically unwell children. The method used for calculating the original SOFA score and the pSOFA score was the same [8]. Each system was given a subscore, which ranged from zero to four points, based on the variable that performed the worst throughout each 24-hour period. A daily pSOFA score, which ranges from zero to 24, is produced by adding the six subscores for each 24-hour period. Higher scores denote a worse outcome. Consistent with the original criteria, a variable was deemed normal if it was not measured within a 24-hour period. The PELOD score, the updated PELOD2 score, and the Pediatric Multiple Organ Dysfunction Score were the three additional pediatric organ dysfunction scores against which the performance of the pSOFA score was tested. In the subgroup of patients with suspected or confirmed infection as well as in the general PICU population, the pSOFA score demonstrated excellent discrimination for in-hospital mortality. Wiens et al. conducted study which was among the first to estimate, in a typical resource-constrained situation in Africa, the burden of non-neonatal pediatric sepsis in children with suspected infection using the international standard criteria of sepsis [13]. Given that sepsis was present in the majority of children with illnesses, this criterion was found to have relatively low specificity but excellent sensitivity in identifying those who died. In this context, the definition of sepsis based on the SIRS is not very useful in identifying children who are at high risk of dying in the hospital.

Baloch et al. conducted an evaluation comparing the diagnostic precision of the PRISM3 score and pSOFA in predicting mortality among critically ill children [5]. Children who did not survive exhibited significantly higher pSOFA and PRISM scores compared to survivors. When predicting the 30-day mortality, a pSOFA cutoff value greater than two demonstrated a sensitivity of 93.87%, a specificity of 38.21%, and an accuracy of 69.93%. In contrast, a PRISM3 24 score cutoff value greater than eight showed a sensitivity of 55.83%, a specificity of 77.24%, and an accuracy of 65.03%. The findings suggest that the pSOFA score serves as an effective predictor for the 30-day mortality in critically ill children, outperforming the accuracy of the PRISM3 24 score. Weiss et al. conducted an evaluation of the applicability of the pSOFA score in the emergency department (ED) setting [4]. They found that a pSOFA score of two or higher was infrequent but correlated with increased hospital mortality, although it demonstrated poor sensitivity as a screening tool for hospital mortality. In contrast, children with a pSOFA score of two or less exhibited a minimal risk of death, showcasing high specificity and negative predictive value. Particularly among patients with suspected infection, those identified with pSOFA-defined septic shock exhibited the highest mortality.

In a study by Zhao et al., it was noted that the pSOFA score outperformed the SIRS in the diagnosis of infected children with a heightened risk of mortality [10]. In a study by Dewi, the pSOFA score, assessed on admission day, as well as on days 2, 4, 7, and 14 in the PICU, proved superior in predicting prognosis for pediatric oncology patients on mechanical ventilator and pediatric patients with sepsis [14]. It also exhibited better accuracy in predicting the 30-day mortality compared to other scoring systems. However, the pSOFA score did not effectively predict the length of stay for pediatric patients in the PICU.

Zamir et al. reported that the pSOFA score displayed a sensitivity of 88.9% on day 0, which increased to 100% by day 7 in predicting mortality [15]. Specificity was 80.6% on day 0 and 97.0% on day 7 [15]. The diagnostic accuracy of pSOFA in predicting mortality was 81.6% on the day of admission, escalating to 90.8% on day 3, and reaching its highest accuracy on day 7 at 97.4%. Consequently, the pSOFA score demonstrated robust diagnostic accuracy in predicting mortality in pediatric patients admitted to intensive care, with increasing accuracy observed over the duration of the stay.

In an exploratory analysis by Paulsen et al., combining the 2005 Goldstein criteria, the institutional sepsis screening tool (ISST), and pSOFA into a hybrid screening model exhibited superior sensitivity (84.3%) and outcome discrimination [16]. The pSOFA score showed noninferior sensitivity compared to a Goldstein-based institutional sepsis screening model and emerged as a superior discriminator of poor clinical outcomes. Ferreira et al. revealed that the nonsurvived cases had a considerably higher pSOFA score (13.5, 5.5, respectively) than the survived cases [17]. In terms of death prediction, total pSOFA of ≥9.0 had highest diagnostic characteristics and reported that the pSOFA score is a valid predictive indicator for PICU patient death.

Sepsis poses a prevalent challenge in all healthcare ICUs, significantly contributing to morbidity and mortality, particularly in children on a global scale. While comprehending a patient's risk of mortality is undeniably crucial, what holds greater significance for clinicians is the capacity to precisely forecast an individual patient's trajectory in the days following admission to the PICU. Identifying patients at imminent risk of deterioration is paramount, as vigilant monitoring enables prompt and effective interventions, ultimately enhancing patient outcomes [18].

Addition of various parameters to pSOFA to increase predictive value

In order to predict the mortality of critically ill children, Gorla et al. assessed the viability of adding adjunct echocardiographic parameters to the pediatric version of the SOFA score (pSOFA-E score), adapting and validating it in relation to the pSOFA score [19]. They found that the addition of a score created by requiring ionotropes to maintain an adequate ejection fraction defies simple bedside echocardiography to the pSOFA score, which is highly beneficial and accurate in differentiating between PICU mortality, morbidity, and cardiovascular status/compromise of the body. Kumbar et al. also reported that an increase in pSOFA-L score is associated with high mortality and poor outcome [20]. The findings of the present study validate and emphasize that pSOFA-L score helps in the accurate prediction of mortality of critically ill children. In the general PICU population, El-Mekkawy et al. evaluated the predictive usefulness of lactate in comparison with the pSOFA score [21]. For predicting mortality, a 24-hour lactate level and pSOFA are also helpful. It was found that the 24-hour lactate level added to the pSOFA score improved its predictive value. Additionally, Maheshwari et al. discovered a strong correlation between the pSOFA-L score and serum lactate levels, with greater lactate levels suggesting a worse prognosis [22].

## Conclusions

An ideal scoring system in hospitals should be accurate, easy to use, and low cost. However, no scoring system is flawless, and ongoing studies aim to improve accuracy and develop new tools. The pSOFA score accurately determines mortality in pediatric patients receiving critical care. It is useful for predicting the 30-day mortality in the general PICU population and demonstrates better accuracy than the PRISM3 24 score. The SOFA score, employed at admission, is valuable for predicting outcomes in the general PICU population and is more accurate than SIRS for defining pediatric sepsis.
